# Deep Reinforcement Learning Environment Approach Based on Nanocatalyst XAS Diagnostics Graphic Formalization

**DOI:** 10.3390/ma16155321

**Published:** 2023-07-28

**Authors:** Dmitry S. Polyanichenko, Bogdan O. Protsenko, Nikita V. Egil, Oleg O. Kartashov

**Affiliations:** The Smart Materials Research Institute, Southern Federal University, 178/24 Sladkova, 344090 Rostov-on-Don, Russia; bprocenko@sfedu.ru (B.O.P.); ntoporkov@sfedu.ru (N.V.E.); okartashov@sfedu.ru (O.O.K.)

**Keywords:** graphical representation, digital model, X-ray absorption spectroscopy, functional decomposition

## Abstract

The most in-demand instrumental methods for new functional nanomaterial diagnostics employ synchrotron radiation, which is used to determine a material’s electronic and local atomic structure. The high time and resource costs of researching at international synchrotron radiation centers and the problems involved in developing an optimal strategy and in planning the control of the experiments are acute. One possible approach to solving these problems involves the use of deep reinforcement learning agents. However, this approach requires the creation of a special environment that provides a reliable level of response to the agent’s actions. As the physical experimental environment of nanocatalyst diagnostics is potentially a complex multiscale system, there are no unified comprehensive representations that formalize the structure and states as a single digital model. This study proposes an approach based on the decomposition of the experimental system into the original physically plausible nodes, with subsequent merging and optimization as a metagraphic representation with which to model the complex multiscale physicochemical environments. The advantage of this approach is the possibility to directly use the numerical model to predict the system states and to optimize the experimental conditions and parameters. Additionally, the obtained model can form the basic planning principles and allow for the optimization of the search for the optimal strategy with which to control the experiment when it is used as a training environment to provide different abstraction levels of system state reactions.

## 1. Introduction

The proposed study aims to automate and accelerate the process of new functional nanomaterial diagnostics using advanced instrumental methods. The X-ray absorption spectroscopy (XAS) data allow us to evaluate the qualitative and quantitative characteristics of materials with high accuracy. Synchrotron centers are some of the most important facilities for experimental research in the field of materials science. However, the use of synchrotron radiation is strictly regulated in terms of time, which forces researchers to make prompt decisions about the series of experiments to be conducted. To facilitate this process, the proposal is to use a coalition of artificial intelligence agents that are capable of the real-time optimization of the input parameter sets of experiments, the screening and control of the course of experiments in a series, and a preliminary assessment of the results obtained; this will significantly reduce the resource costs and increase the efficiency of synchrotron experiments.

Deep reinforcement learning (DRL) technologies have proven their effectiveness in complex system planning and control compared to other optimal control methods for many applications [1,2,3,4,5]. Real-time optimization solutions can be included in manufacturing processes [6], complex physical problems [7,8,9] where alternative approaches are computationally inefficient, and, as in our case, the optimal planning of experimental studies [10]. There are deep reinforcement learning approaches that do not require a virtual training environment to control complex systems. For example, in [3], the authors proposed the use of primitive environment models followed by a tweaking of the policy gradients based on historical target parameter data. However, the scalability of such a solution is too weak to be applied in the multiscale physicochemical system context. On the other hand, learning approaches based on different kinds of models have several advantages. These include reduced resource costs for the collection of expensive data, reduced computational complexity in optimizing agent policies for high-dimensional multiscale systems, and the ease of scaling this approach in a multiagent learning framework [11,12,13]. The solution could be a virtual space for the real descriptors of the physical environment of the experiment; this would conditionally provide a digital model of the processes taking place during the experiment and would be a reliable computer model of the learning environment for DRL agents. There are no suitable digital models of the experimental physical environment of nanomaterial diagnostics based on synchrotron radiation with which to solve these problems. In essence, the task of the agents in our case is like the task of searching for an optimal trajectory in an uncertain multidimensional space of actions and states, the solution to which requires a reliable physical environment, reward function, and goal space [2]. The key difference in our case is the conditional digital model of the physical experimental environment, which forms its own multidimensional space of actions and states; otherwise, we can extrapolate the numerous experiences of researchers dealing with DRL problems [4,5,14,15,16,17].

In general, the development of reliable digital models of objects, processes, and entire systems can be considered as a joint task with the model-based approach to DRL; it can also be considered independently. Unfortunately, to date, there is some bias in this area toward the creation of digital models and digital twins of manufacturing processes and enterprises in general. However, the results of the practical integration allow us to extrapolate this experience to our study. It is often difficult to plan experiments, and it is difficult to find a suitable laboratory and suitable equipment. The subsequent analysis and interpretation of the results takes an enormous amount of time and resources. Because of this, the experiments are limited and quite slow to contribute to the disclosure of the scientific idea as well as to integrate the obtained scientific results into the real sectors of the economy and social life of society. These factors determine the importance of creating digital models of experimental environments for the reproducibility of scientific results, developing strategies for the optimal planning of experiments, and obtaining new scientific results much faster. These issues are not new and have been considered for the past decade [18]. During this time, the approaches to creating digital models and digital twins of complex multiscale systems have proven their effectiveness [19,20,21]. The key factors in the multiscale systems of digital model design are reliability, determined by validation and verification [22]; the definition of valid interfaces and links to the experimental process [23]; the determination of the final model’s details at the stage of formalizing the complex system’s representation [24].

In our case, the metamodel-based graphical representation for the physical experimental environment of a palladium nanocatalyst’s diagnostics requires the formalization of the instrumental part of the XAS methodology. The following sections are devoted to this. The rest of this structure is organized as follows. Section 2 is devoted to the description of the process of the experimental investigation of local atomic and electronic structures of palladium nanocatalysts using synchrotron radiation. In Section 3, a methodology is proposed for the representation of physical experimental environments as a multiscale system with a finite number of components and connections between them. Section 4 includes descriptors of the experimental system’s main physical and chemical entities as well as algorithms for the optimization of the graph’s topological structure, the validation and verification of its structure, and the effective representation of the system states. Section 5 reveals the main results in a diagram of the components of the digital model of the physical experimental environment for palladium nanocatalyst diagnostics; the “Section 6” represents the main advantages and limitations of the study and discusses future work; the “Section 7” contain the main outcomes of the study in a graph formalization of a complex multiscale physicochemical experimental environment.

## 2. Related Work

XAS is a highly versatile and powerful analytical technique used across a vast range of scientific disciplines including materials science, chemistry, and biology to elucidate the electronic and local atomic structure of matter [25,26,27,28,29,30,31,32,33,34,35]. As knowing the features of the material’s structure provides valuable insight into its structure–property relationships, XAS is a valuable tool for the study and fine-tuning of various materials, especially catalysts [29,36]. These materials dramatically speed up the chemical reactions they are involved in, providing an alternative reaction pathway with lower activation energy and thereby drastically facilitating reaction yield and selectivity [26,36,37,38,39]; the vast majority of industrial chemical processes utilize catalysts [40].

In catalysis, the reaction mechanism and nature of the active sites, along with the changes the catalyst can undergo during the reaction, are of the highest importance for the design of efficient catalysts [25,31,40,41,42]. Consequently, in situ XAS is often used to characterize the catalyst’s structural evolution and the local environment of the active sites during both the heterogeneous form, where the catalyst is present in a different phase to that of the reactants, usually as a solid catalyst in a gas or liquid reaction mixture, and the homogenous version, which, in turn, involves a catalyst in the same phase as the substrate, most commonly a transition metal complex in a liquid solution [40]. The great advantage of XAS over other techniques such as X-ray diffraction (XRD) is that XAS is an element-specific technique [28,29], which can be used in operando and in situ regimes [38,43,44], and it is sensitive to both the surface and bulk [39,44,45] of the studied catalyst.

**Figure 1:**
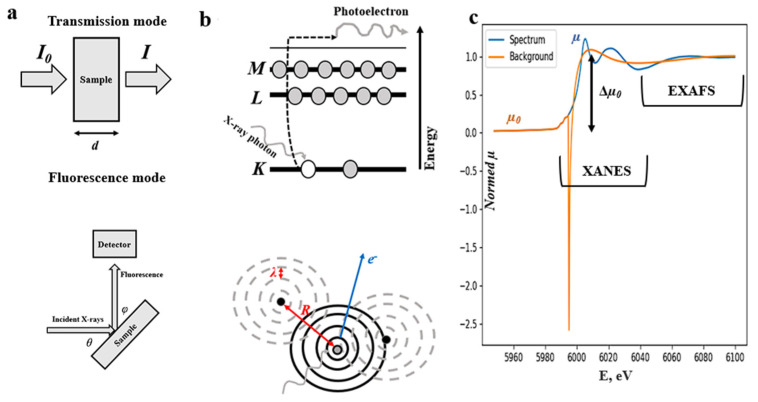
Scheme of the possible experimental geometries (**a**); the graphical representation of the physical principles underlying the XANES (**top**) and EXAFS (**bottom**) parts of XAS spectrum (**b**); and typical XAS spectrum (blue) regions with smooth background (orange), usually subtracted from the spectrum during EXAFS analysis (**c**).

When XAS spectra are collected, a beam of X-rays is directed toward a sample and either the amount of X-ray energy passed through the sample is measured, as in the case of the transmission mode technique, or, for the fluorescent mode, the intensity of fluorescent radiation, which the sample emits upon being excited by high-energy X-rays, is determined (Figure 1a) [28,29,35]. The latter is preferable when the X-ray beam energy is not sufficient to penetrate the sample or if the sample is too thick or thin. Although great progress has been made in the field of laboratory XAS [27], synchrotron radiation remains the best choice for the source of X-rays due to the synchrotron radiation brightness, wider energy range, and better spectral resolution [25].

The absorption coefficient *µ*(*E*), which is of particular interest in XAS, can be calculated from the Beer–Lambert law as a function of incident radiation energy *E* as

(1) $$\mu \left(E\right)=\ln\left(\frac{I_{0}}{I}\right),$$

in the case of the transmission technique and with the use of the equations

(2) $$I_{f}=I_{0}\frac{\varepsilon \omega }{4\pi }\frac{\mu_{\chi }\left(E\right)\left[1-e^{-\left(\frac{\mu_{tot}\left(E\right)}{\sin\left(\theta \right)}+\frac{\mu_{tot}\left(E_{f}\right)}{\sin\left(\phi \right)}\right)d}\right]}{\frac{\mu_{tot}\left(E\right)}{\sin\left(\theta \right)}+\frac{\mu_{tot}\left(E_{f}\right)}{\sin\left(\phi \right)}},$$

and

(3) $$\mu_{tot}\left(E\right)=\mu_{\chi }\left(E\right)+\mu_{other}\left(E\right),$$

for the fluorescent mode measurements, where *µ_tot_* is the total sample absorption; *µ_χ_* is an absorption of the chosen element; *θ* is an angle between the incoming beam and the sample surface; *ω* is the solid detector angle; *ϕ* is an angle between the fluoresced radiation and the surface of the sample; *ε* is the efficiency of the fluorescence; d is the sample thickness; the values *I*_0_, *I*, and *I_f_* state the intensity of the incident, as passed through the sample and fluoresced radiation, respectively. Equations (2) and (3) are more sophisticated compared to Equation (1) due to the self-absorption of the sample [26,29]. In practice, simpler versions of Equation (2) are used and assume cases of thick or thin samples [28,31].

The X-ray absorption spectroscopy is based on a quantum-level description of the interaction of X-rays with matter [25,28,31,46]. When an incident high-energy photon is absorbed by a core-level electron within the atom, the latter can be ejected from the atom in the form of the photo-electron (Figure 1b), which results in a sharp absorption edge in the X-ray absorption near-edge structure (XANES) part of the XAS spectrum (Figure 1c), which usually occupies 30–60 eV beyond the absorption edge [25,46]. Then, the ejected electron can scatter (Figure 1b) from the neighboring atoms before recombination, giving rise to the extended X-ray absorption fine structure (EXAFS) region, which usually spans over 100–200 eV beyond the XANES region (Figure 1c) [47].

Analysis of the extended X-ray absorption fine structure data can provide the interatomic distances, coordination numbers, and, therefore, the structure of the active metallic center and bonded ligands of the homogeneous catalysis or the surface and bulk structure of the heterogeneous catalysis [26,28,42,48]. Usually, EXAFS processing is performed by fitting the well-known EXAFS equation

(4) $$\chi \left(k\right)=\sum_{j}\frac{N_{j}e^{-2k^{2}\sigma_{j}^{2}}e^{-2R_{j}/\lambda \left(k\right)}f_{j}\left(k\right)}{kR_{j}^{2}}\sin\left(2kR_{j}+\delta_{j}\left(k\right)\right),$$

where *N_j_* is the absorbing atom coordination number of the *j*-type atoms; *R_j_* is the distance to them; *σ*^2^ is the Debye–Waller factor, which is proportional to the disorder of the *j*-atom positions; *f_j_*(*k*) and *δ*_j_(*k*) are the scattering properties of the *j*-atom, which are usually obtained by means of multiple-scattering theory calculations in codes such as FEFF [49]; χ(k) is a value connected with the aforementioned *µ*(*E*) in the manner described by the following equations

(5) $$\chi \left(E\right)=\frac{\mu \left(E\right)-\mu_{0}\left(E\right)}{\Delta \mu_{0}\left(E\right)},$$

(6) $$=\sqrt{\frac{2m\left(E-E_{0}\right)}{\hbar^{2}}},$$

where *µ*_0_(*E*) is a smooth background function for an EXAFS part of the spectrum (Figure 1c); Δ*µ*_0_(*E*) is a jump in absorption of the XAS spectrum; *m* is a mass of an electron; ℏ is the Planck constant; *k* is a wavenumber; *E*_0_ is an absorption edge energy [28,29].

Unlike EXAFS, the interpretation of XANES data is not straightforward since there are no explicit physical descriptions. However, great results are achieved using the fingerprint approach together with ab initio calculations [29,46,50], chemometrics-inspired methods, namely, multivariate curve resolution (MCR) and principal component analysis (PCA) [51,52,53], or even machine learning-supported techniques to reveal the formal charge and coordination environment of the metal complex or to study the charge state of the nanoparticle [45,54,55,56].

Although the field of XAS for nanomaterial characterization is constantly evolving, a few hot topics have been especially prominent in the last decade. The main aspects of the growing complexity of the on-the-fly catalyst X-absorption spectroscopy characterization, together with the expanding amount of data obtained, pose the problem of autonomous data acquisition with real-time active feedback control over the experimental protocol, which can be addressed by machine learning or deep reinforcement learning (DRL) algorithms [57].

## 3. System Functional Decomposition

In order to use and train the DRL agents controlling the experimental process, the given system should first be represented as a ‘black box’ digital model, consisting of a finite number of components with determined relationships between them (i.e., decomposed to the interacting collection of subsystems organized hierarchically at multiple levels) [58].

The functional decomposition paradigm was chosen [58] by considering the functional nature of the desired digital model, where a number of input parameters were mapped to multiple output observables, and a few independent variables were also involved. As additional guidance, the spatial and hierarchical decomposition principles were introduced. Whilst hierarchical principles provide the generality and better flexibility of the model, the spatial principles separate the system constituents of a different physical nature [59] (vide infra).

Based on the aforementioned principles and their main task, the model of the experimental system was represented by a mapping from the quantities, which can be varied during the experiment, to the observables, which can be detected during the experiment. At the same time, the various parameters, which are intrinsic to the specific experiment, should be considered as constants. As input values, the system temperature and reactant flows can be determined, and the observables are divided into two main groups: ‘physical’, represented by the XAS spectrum, and ‘chemical’, which can be described in terms of chemical reaction engineering, which employs the terms ‘yield’, ‘conversion’, and ‘selectivity’ as ratios to describe the amount of reactant used (conversion) and the amount of desired product generated (yield) compared to the unwanted product (selectivity); these terms are denoted by X, Y, and S, respectively [40]. The experimental constants, in turn, can be represented as the type of catalyst (local atomic structure of molecule employed or surface morphology, nanoparticle size, and type of support surface of the heterogeneous catalyst) and the measuring setup features (cell geometry).

For a homogeneous catalytical system, it is reasonable to implement multiscale representation, where the entire fluid or liquid system is described as a continuous medium; for example, the simulation of the fluid dynamics, the finite element method and the reaction kinetics [60,61], and the corresponding ‘chemical’ output are modeled at the atomic level. In the end, fine quantum-chemical phenomena, which determine the properties of the atomic-level simulation and the systems’ XAS response, can be implemented as ab initio self-consistent field (SCF) calculations [49,62,63,64,65,66,67]. The main intrinsic properties for each subsystem can be expressed as follows: velocity and temperature tensor fields (fluid level), interatomic potential fine structure (molecular level), and chemical and local atomic structure of the single catalyst molecule (quantum level).

In the case of heterogeneous catalysis, the catalysts and reactants can be separated into the ‘reaction mixture’, ‘nanoparticle surface’, ‘bulk structure of nanoparticle’, and ‘catalyst support’ subsystems. The first is characterized by the partial pressure of the reactants, with the total pressure being their algebraic sum. The second one, characterized by the coverage value, is responsible for the ‘chemical’ part of the model output, possibly by means of analytical surface reaction models or microkinetic modeling; this involves the kinetic Monte Carlo (KMC) [68,69,70] or molecular dynamics (MD) [71,72,73,74] simulations and is affected by the ‘reaction mixture’ and ‘bulk structure of the nanoparticle’ and can affect the resulting XAS spectrum in the case of a small nanoparticle. The ‘bulk structure of nanoparticle’ system is characterized by a charge state and interatomic distances and defines both the surface reaction parameters and the XAS spectrum. The latter can be calculated using the finite difference method or modeled by the deep learning surrogate model [63,65]. ‘Catalyst support’ strongly affects the bulk part of the nanoparticle through charge flow and strong support interaction, which can be computationally determined easily and effectively by density functional theory (DFT) calculations [62,75]. The total pressure and temperature, along with the cell geometry, strongly affect the coverage and, therefore, the chemistry part of the whole model output [60].

## 4. Graph Modeling and Structure Optimization

**Figure 2:**
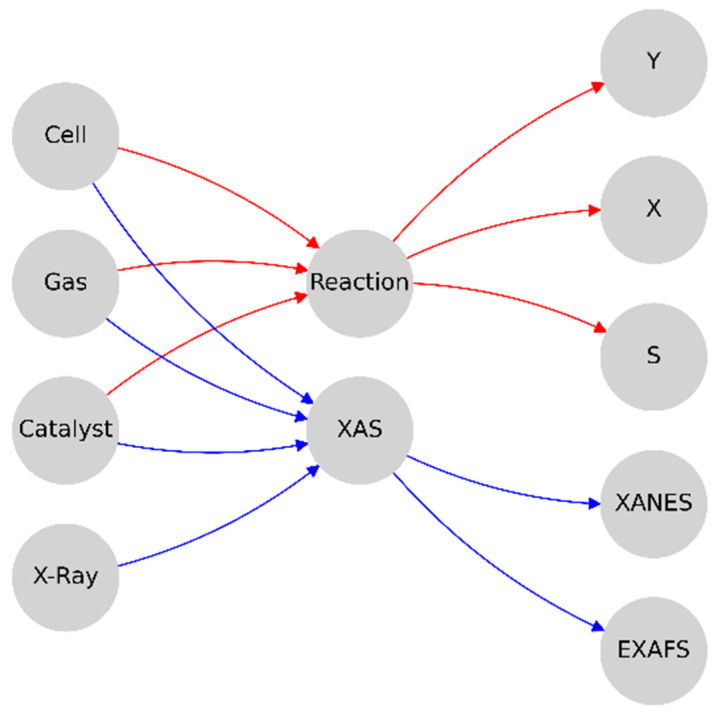
A primitive schematic representation of the system with three levels—input parameters, methods, output results.

The development of a graphical representation of the physical experimental environment was carried out in several steps. At the first stage, the conditional division of the available system components into the corresponding input conditions, mutual influences, and related parameters, together with the computing modules and output elements, was performed; this dealt with the system responses in the format of synthetic spectra and key diagnostic results (Figure 2).

For further consideration, the components of the system descriptions for all the physical entities used in the graph representation are listed in Table 1, with the data types and structures denoted. The latter part describes the mathematical and informatic nature of each element of the functional decomposition.

The main data types used were integer variables, floating point variables, and univariate and multivariate data arrays. In addition, for convenience, the individual physical entities represented by the gradient distribution of the magnitude in the general form were replaced by table approximations. It should be considered that in the case of a homogeneous catalyst, its molecular structure represents the main node, which defines the «Electronic structure» and «Interatomic distances», which in turn directly affect the course of the reaction and the XAS spectra. With a heterogeneous catalyst, the atoms of the catalyst can be assumed to be distributed over the support («Catalyst support»), and the combination of support and catalyst types determines the electronic and atomic structures of the catalyst, for which «Catalyst» defines the values of «Electronic structure», «Interatomic distances», and «Charge State» nodes.

**Figure 3:**
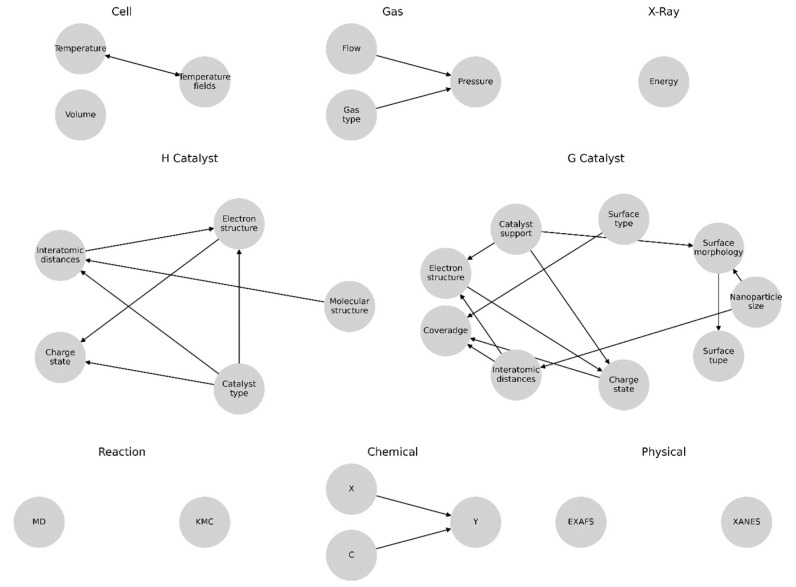
Graph representation modeling the individual components of the decomposed system.

For all the elements that describe the input parameters and measurement conditions, the subgraphs were formed independently, reflecting the high variance of the possible parameters for the two main cases: homogeneous and heterogeneous catalysis (Figure 3).

An intermediate analysis showed that for the homogeneous catalysis, one of the most important parameters was represented by molecular structure, from which the nodes corresponding to the electronic structure («Electric structure») and the interatomic distances of the sample («Interatomic distances») followed and directly affected the course of the reaction and the XAS spectra. For the heterogeneous catalysis, it was assumed that the clusters of the catalyst’s active centers were uniformly distributed over the support («Catalyst support») and that the combination of the carrier and catalyst types determined the electronic and atomic structures of the catalyst, according to which the catalyst affected the values of the electronic structure («Electronic structure»), interatomic distances («Interatomic distances»), and charge state («Charge state») through the connections.

**Figure 4:**
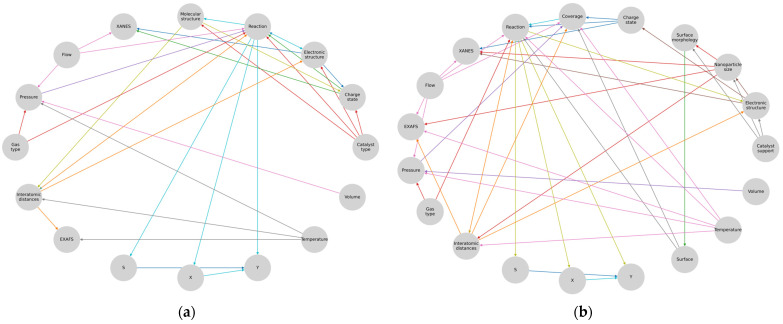
Formalization of the experimental systems of (**a**) homogeneous and (**b**) heterogeneous catalysis.

The analysis of this representation allowed us to make a few simplifications. We removed X-rays from consideration since they only affected the energy region of the catalyst under study, which depended on the electronic configuration of the atoms and did not affect the other regions of the model. The activation energy could also be omitted since it was already included in the calculation of the reaction and was a constant value. The temperature effect of the X-rays and the reaction effects (endo-/exothermal) could also be excluded from consideration, since the temperature of the sample was usually controlled. The next simplification was the combination of temperature as a single quantity and the temperature variable as a scalar field into one object. In principle, the temperature field can be removed, since most experiments take place in cells with a small volume. Such an approach is possible only if the molecular dynamics and the kinetic Monte Carlo simulations are integrated together, or if only one of them is used. Furthermore, the homogeneous (Figure 4a) and heterogeneous (Figure 4b) systems can be considered separately in the graph representation.

**Figure 5:**
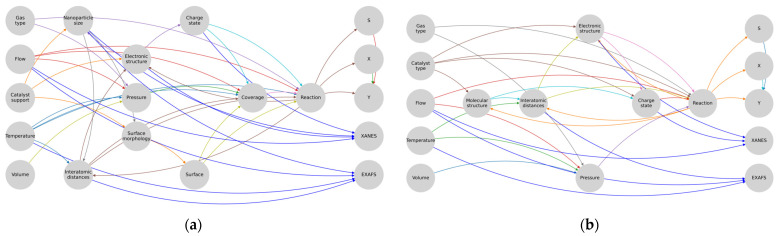
Subgraphs of (**a**) heterogeneous and (**b**) homogeneous catalysis.

Next, we can represent these representations in a more convenient way, where the results are on the right side and the unique diagnostic parameters that cannot be computed from the others are on the left (Figure 5).

Here, the connections leading to the XAS are highlighted with a single color since these are the parameters that directly influence the spectrum or can be calculated from it and that in turn are influenced by the parameters and processes in the «reaction» domain of the model representation. The next step was to arrange and merge all the graph structures obtained in the previous steps into one. To optimize the obtained metamodel, the author’s Algorithm 1 was developed, taking the input data stream describing the graph and forming unique user indices based on its nodes.
**Algorithm 1.** Compressing Repeating Graph Nodes**INPUT:** x node coordinate, y node coordinate, name of node, input edges, output edges **OUTPUT:** Custom list with nodes and edges. nodes = [] Indexes = 0 uniq_index = {} for i in inputStream():  is_uniq = list(filter(lambda x: i[2] in list(inputStream()))) support_in = []  support_out = []  for j in range(i[7], len(i)):    if j <= i[5]:      support_in.add(j)    else:      support_out.add(j)  if is_uniq:    uniq_index.add(indexes, i[2])    nodes.add(indexes, i[2], i[3], i[4], i[5], i[6], support_in, support_out)    indexes+ = 1  else:    nodes.add(list(uniq_index.keys())[list(uniq_index.values()).index(i[2])], …, i[2], i[3], i[4], i[5], i[6], support_in, support_out) graph = [] for i in range(len(nodes)):  for j in range(i + 1, len(nodes)):    if nodes[i][0] == nodes[j][0] and nodes[i][0] != None:      nodes[i][6].extend(nodes[j][6])      nodes[i][7].extend(nodes[j][7])      nodes[j][0] = None for I in nodes: if i[0] != None  graph.add(i) return graph

**Figure 6:**
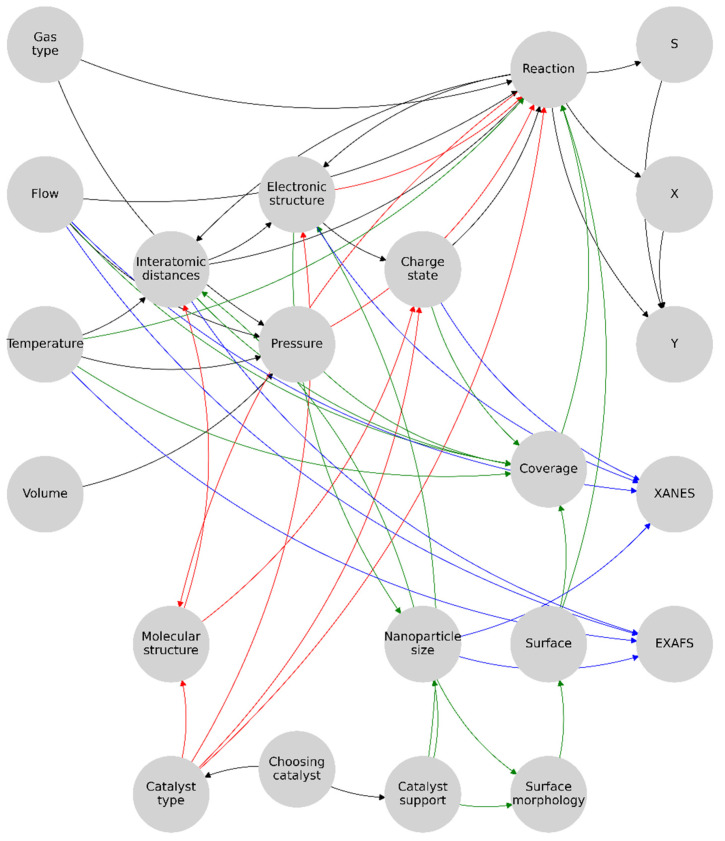
Proposed graph structure formalizing an experimental diagnostic system for palladium nanoparticle-based catalysts. Here, the main or general part of the graph representation of the system is highlighted in black, the active links in the homogeneous catalyst are in red, the active links in the heterogeneous catalyst are in green, and the parameters directly affecting the XAS are in blue. In total, 32 bonds are needed to simulate the reaction diagnostics of homogeneous catalysis and 42 are needed for the heterogeneous catalysis; the total representation model contains 51 bonds.

As the functionality of this algorithm is to preprocess and optimize the graph structure, there is no need for complex keys, and the unique node can be indexed by a single integer number. Then, a collection of nodes is formed, where each contains the index, node name, *x*, *y*, the number of incoming edges, the number of outgoing edges, the collection of incoming nodes, and the collection of outgoing nodes. Taking into account the fact that even though some nodes were the same, they may have had different edges, so we collapsed the same nodes into one and found repetitive indices and thereby handled the content of the nodes. We left *x* and *y* of the first one and merged the edges and nodes and removed the repeating ones. Thus, we obtained a convoluted graph of only unique nodes as a user-defined complex dictionary. This made it possible to identify the nodes common to the homogeneous and heterogeneous graphs and to correctly interpret their contents from the conditions of the elements of the experimental system. In essence, the catalyst-type independent part of the model was optimized. Next, the parameters unique to each catalyst type, the catalyst type, and the catalyst support were identified. Selecting one of these parameters determined the type of reaction within the model representation. The final version of the graph representation of the experimental system is shown in Figure 6.

At the final stage, the author’s Algorithm 2 was implemented to validate and optimize the metamodel graph representation of the physical experimental environment, based on real data from the instrumental studies of palladium nanocatalyst diagnostics using synchrotron radiation. The general approach was to compress and analyze the potential number of states of the graph structure of the physical medium. The implementation of this algorithm used the traditional approaches to breadth-first search with modifications for sorting and merging with multiple outputs. A special feature was the organization of the algorithm with the domain knowledge of the type of catalytic reaction being modeled and the comparison with real scenarios of similar systems. This approach allowed us to confirm the physical validity of the graph metamodel and to carry out validation of the selected computational methods of the individual elements of the system.
**Algorithm 2.** Sorting and merging in width with multiple output**INPUT**: custom graph’s data **OUTPUT**: processed graph’s data upper = [] downer = [] next = [] upper.add (graph[0]) downer.add (graph[0]) next.add (graph[0]) def disable_nodes(result_graph, graph):  for node in graph:    if list(filter(lambda x: node[0] in list(result_graph))) and node[7] == None      result_graph = remove(node, result_graph)   return result_graph def adjust(list):  result = []  for I, j in enumerate(list):    if I == 0:      result.append ((None, list[i + 1]))    elif I == len(list)−1:      result.append ((list[i−1], None))    else:      result.append((list[i−1], list[i + 1]))  return result while True:  nodes = []  for node in next:    if node == downer[len(downer)−1] and node != None:      upper.add (node)    for i in adjust(node):      nodes.push_back(i)  temp_collection = []  for node in nodes.sorted().uniq():    temp_collection.add(node)  out_next, out_downer = [], []  while downer or temp_collection:    if next and temp_collection:      downer_head = next.peek()      temp_collection_head = temp_collection.peek()[0]        if downer_head == temp_collection_head:          out_downer.add(next.pop())          temp_collection.pop()        elif downer_head < temp_collection_head:          out_downer.add(downer.pop())        elif downer_head > temp_collection_head:          out_next.add(temp_collection_head)          out_downer.add(temp_collection_head)          out_upper.add(temp_collection.pop())        elif downer:          out_downer.add(downer.pop())        elif temp_collection:          out_next.add(temp_collection.peek()[0])          out_downer.add(temp_collection.peek()[0])          out_upper.add(temp_collection.peek()[0])  result_graph = out_downer.extend(out_next) out_graph = disable_nodes(result_graph, graph)



In this algorithm, we adopted the previously developed custom dictionary. We formed collections of states: child and parent, visited, and required to be visited. We prepared a custom width search solution, where the solution step represents a node of our dictionary with all the associated data. We performed sorting and merging with multiple choices to form groups of copies of paths that were completed with information about the visited states. As a result of the processing, we obtained copies of the traversed paths. For the selected data structures, it was possible to disable the null (unused) nodes using the previously created unique index by removing them from the resulting traversal. In addition, since no changes were made to the original data structure during the algorithm’s operation, the node disconnection was possible for any graph pass in real-time, since the changes were only made to the resulting traversal structure.

## 5. Digital Model Design

**Figure 7:**
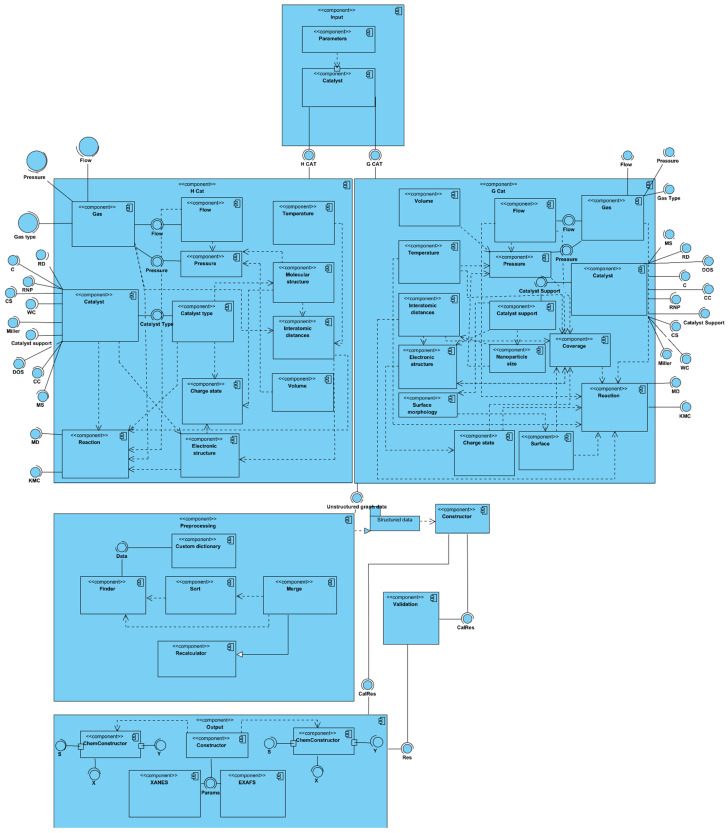
Component diagram of the proposed digital model of the physical experimental diagnostic environment for palladium nanocatalysts.

Based on the resulting graph representation model, a UML diagram of the components of the experimental system was developed. It interpreted the future software structure of the digital model of the physical experimental environment and presented an abstract encapsulation of the classes, regulating all the relationships between the main elements of the proposed model software implementation of the model (Figure 7).

According to the developed graph, a component diagram describing the software of the digital model was implemented. The resulting graph was divided into two main groups of components since the type of catalyst used significantly affected the number of nodes and links of the graph and, consequently, the number of states and the length of the pass. We also added the developed algorithms for the data preprocessing, search, sorting, and merging as well as a validation component. In general terms, the component diagram presented is described as follows:

1. Input component. This represents a simple set of software structures that are needed to form the input parameters. Depending on the type of catalyst, one of the two main components of the diagram will be performed.
2. Homogeneous catalyst component. This contains the interpretation of the linear graph. The main subcomponents contain the requirements for the input data, which are represented as interfaces that require implementation. All of the requirements are fulfilled based on the input data package from the input component. Some interfaces are converted into separate subcomponents after collection and processing to enable additional operations. Furthermore, dependency lines are drawn between all the parts according to the original graph. The conditional output is an unstructured graph, partially containing data that can be computed during the execution phase of the component.
3. Heterogeneous catalyst component. This is structurally the same as component 2, but it contains additional components and link lines according to the original graph.
4. Pretreatment component. This contains all the solutions for creating and processing a custom dictionary of states. In addition, this step should perform the search, sort, and merge functions with multiple choices. Depending on the type of catalyst and the compiled pass, the null nodes of the graph are disconnected. The output in this component is a package of processed and structured data that is suitable for both algorithms and the deep learning model.
5. Output component. This contains the main component, called the constructor, which defines the type of computation (chemical or physical) and, depending on the type, transmits data for computation.
6. Validation component. This contains a set of algorithms that calculates the validity function and compares the obtained data with the results of the deep learning model. It is an optional component that must be disabled after obtaining stable qualitative results from the model.

The diagram is formed in such a way that each component can be represented in two ways: (1) as a set of classical algorithms with a class structure or (2) as a model of machine learning or deep learning. Thus, each component can be replaced by an artificial intelligence algorithm at some point in the software implementation without losing the dependencies and requirements. This approach allows for the implementation of a convenient black box system with a hot swapping capability. With the help of the developed component program, it is possible to implement flexible and scalable software, which will be versatile and reliable in the context of the task performed.

## 6. Discussion

The implementation of RL agents for the experimental design optimization and control can drastically facilitate material diagnostics, leveraging a compromise between exploration and the exploitation of the experiment’s parameter space, especially in the case of the in situ and operando regimes. By taking into account the XAS diagnostics of palladium-based nanocatalysts in these regimes, it was shown in several works [39,76] that both the reversible and the irreversible simultaneous formation of multiple impurity phases can occur under reaction conditions. The same can also be said about the other systems [77,78]. These cases illustrate only one of the sources of complexity that represent a challenge in system exploration and characterization, and DRL can be one of the promising solutions for all of them [6,7,8,9].

The metagraph representation approach for building digital models presented in the work utilized the same ideas of physical system decomposition that are usually used in the field of the multiscale modeling [79,80] including the modeling of nanomaterials [81], although the whole model structure is rarely analyzed in terms of the interconnection of the model constituents (i.e., the model decomposition topology). The construction of reliable multiscale modeling frameworks and RL environments requires accuracy and consistency in all the level descriptions, although for the latter, the computational consistency and module-based flexibility is also required [82], especially in the case of deep learning agents. We anticipate the building of a number of solutions for the construction and optimization of multiscale models based on a similar approach; the presented contribution shows only one narrow example of domain knowledge-driven decomposition and metagraph representation of the diagnostics of the XAS nanocatalyst to address that conundrum.

Considering domain-driven physical decomposition, as a preliminary stage of metagraph representation construction, there are a number of other interesting cases whose physicochemical peculiarities require additional effort to be formalized. Going beyond the assumed homogeneous/heterogeneous catalyst dichotomy, single site catalysts combine the unique features of heterogeneous and homogeneous catalysts [83] due to the tunable, unsaturated configuration of the active centers and the strong interaction with the support. Among the existing local probes, XAS has no limitations on the atomic configuration and can be applied in situ to differentiate the charge state, coordination number, and ligand environment of these kinds of catalysts [84]. Another hot topic, where the same approach may face a barrier, is polyoxometalates (POMs), which have been proven to be highly efficient catalysts [85], and for which Brønsted/Lewis acidity and the corresponding redox properties can be tailored for a wide range of relevant applications [86]. Moreover, their properties can be enhanced further by introducing 4f heteroatoms into their structures [87]. Despite the great progress made in the field of POM modeling [88], the capacity of POMs to generate dynamic structures, from the nano- to the micrometer scale [89], can significantly complicate multiscale modeling, and therefore RL environment computational efficiency.

The main contribution of this study is the systemic formalization of a multiscale physical and chemical experimental environment with its subsequent decomposition into functionally original physical and chemical elements and the computational topology reconstruction of their aggregate representation according to the actual real-process image representation in the form of a metagraph. This integration allows for a complete and reliable description of the space of the possible computational states and experimental conditions at different detail levels. This approach can guarantee the stability and reliability of data delivery in the form of a formalized and standardized response under any external influence on the virtual environment. This, in turn, will allow deep reinforcement learning agents to optimize their long-term behavioral strategies and to behave rationally under conditions that were not provided when interacting with the digital model of the experiment.

The main limitations of this study are related to the software interpretation of the graph system representation. The developed component set has many dependencies. During the software implementation, all of the presented dependencies will inevitably spawn many strong associative relationships, where each association will need several previously computed parameters. Despite the modular flexibility, the presented solution will have a complex execution algorithm and a convoluted data cycle. Because of this, the software will be difficult to test and modify externally. The next problem is the multiple requirements. These must necessarily be met for the proper operation. Consequently, the system will only be able to perform its functions when fully populated with the input data. This approach also has a rather high level of computational complexity. The search, sorting, and merging of the components load the system because of a large number of single-type direct operations and the complex structure of the user dictionary.

In the future, we wish to reduce the number of dependencies. Based on the developed component diagram, a class diagram will be built. A more concrete representation of the system will allow us to optimize components and partially replace the relationship types. Further practical implementation of the presented approach in the form of full-fledged software is also planned.

## 7. Conclusions

The main aim of this study was to formalize a physically reliable graphical representation of the digital model of the experimental environment of nanocatalyst diagnostics based on synchrotron radiation. This solution had a methodological basis that formalized a complex multiscale physicochemical environment in terms of a finite number of classes describing the mathematical structure of the experimental processes and the interrelated object types; it also reflected the basic instructions for a reliable software implementation of the digital model based on the principles of the instrumental study of the XAS-tested samples of new functional nanomaterials. To achieve the above goal, several tasks were solved. First, we analyzed the available formalizations for the X-ray absorption spectroscopy diagnostics of the nanoparticle-based catalysts, ending the absence of accurate calculation methodologies with acceptable computational complexity in the XANES domain. However, this physical experimental environment can be represented as a system with a finite number of states with respect to the input vector of the parameters; this system consists of a finite number of components defined implicitly and has a certain structure of input and output data. On the basis of this assumption, a functional decomposition of the experimental system of nanocatalyst diagnostics was carried out, resulting in generalized hierarchical representations of the system components, with descriptions of the physical and mathematical essence of each module individually. Based on these representations, a metamodel of a graph representation of the physical experimental environment, consisting of graphs of the individual system components, was implemented. To analyze and optimize this metamodel, algorithms were implemented that included merging the nodes of the graphs of individual system components with a preservation of the orientation of the edges and a transformation of functional dependence at the vertex; in addition, enumeration was performed in width for sorting and merging with multiple outputs. Based on these algorithms, ‘runs’ of the experimental system states were generated to optimize the metagraph structure based on different types of catalytic reactions and to validate the resulting metamodel on the real data of the XAS spectra profiles. The data were taken from the set we presented in [90].

## Figures and Tables

**Table 1:** Representation of the parameters included in each subgraph.

Short Name	Name	Data Type	Comment
Gas			
P	Pressure	float	Pressure generated in the cell
Q	(Mass) Flow	float	Gas flow rate
id1	Gas type	int	Macro- and microparameters of a particular gas; the index specifies the choice of the file with its description
Cell			
Temperature field	Temperature field	3D array [float]	Scalar temperature field
T	Temperature	float	Average temperature
V	Volume	float	Volume as the characteristic of cell geometry
Catalyst			
R_NP_	Nanoparticle size	float	Scalar size of particles on which the catalyst is located
WC params	Surface morphology	array[Tuple[int]], Array[float], Array1[float], float2	The Miller indices of the surfaces, their surface energies, lattice parameters, and the number of atoms to construct a nanoparticle with the Wulff construction method
Miller indices	Surface type	array[Tuple[int]]	Crystal surface indices of the catalyst and corresponding directional vectors
id2	Catalyst support	int	Crystalline structure; id2 specifies the choice of file with its description
DOS	Electronic structure	Array[float]	Electronic structure, given by the density of electronic states DOS(E)
C	Coverage	float	Percentage of catalyst active centers filled with reacting substances
R_d_	Average interatomic distances	float	A number equal to the average distance between catalyst atoms
CS	Charge state	float	Number equal to the average charge of the atom in the catalyst
id3	Catalyst type	int	Molecular/crystalline structure; id3 specifies file selection with its description
MS	Molecular structure	Array[int]	Vector of atom positions in periodic table and their corresponding cartesian coordinates
Reaction			
MD	Molecular dynamics simulation parameters	string	Path to the file with parameters and the part responsible for molecular dynamics, with the following parameters: The surface structure (from surface morphology, R_d_) Reaction pathways and rates (DFT modeling results and rates) Initial conditions (coverage, T, P, Q) Simulation parameters (time step, the number of simulation steps, and the size of the simulation cell)
KMC	Kinetic Monte Carlo simulation parameters	string	Path to the file with parameters and the part responsible for Monte Carlo, with the following parameters: The surface structure (from surface morphology, R_d_) The potential energy function(s). The initial conditions (Q, T, P, etc.) Simulation parameters (box size, time step, thermo- and barostats, etc.)
X-Ray			
W	Incident X-ray beam energy	float	Beam energy
Chemical			
Y	Yield	float	Ratio or percentage of the theoretical maximum amount that could be obtained
S	Selectivity	float	Ratio of the desired product quantity to the total amount of all possible products
X	Conversion	float	Ratio of the moles or mass of the converted reactant to the initial moles or mass of the reactant
Physical			
	XANES	Array[float]	Fine structure near the absorption edge
	EXAFS	Array[float]	Fine structure of the extended part of the absorption spectrum

## Data Availability

The data presented in this study are available on request from the corresponding author. The data are not publicly available due to privacy reasons.
